# Exploratory Study of Fatty Acid Profile in Two Filmy Ferns with Contrasting Desiccation Tolerance Reveal the Production of Very Long Chain Polyunsaturated Omega-3 Fatty Acids

**DOI:** 10.3390/plants9111431

**Published:** 2020-10-24

**Authors:** Claudia Rabert, Karla Inostroza, Silvana Bravo, Néstor Sepúlveda, León A. Bravo

**Affiliations:** 1 Instituto de Ciencias Biomédicas, Facultad de Ciencias de la Salud, Universidad Autónoma de Chile, Sede Temuco 4810101, Chile; claudia.rabert@uautonoma.cl; 2 Laboratorio de Producción Animal, Facultad de Ciencias Agropecuarias y Forestales, Center of Biotechnology on Reproduction, Scientific and Technological Bioresource Nucleus Universidad de La Frontera, Casilla 54D, Temuco 4811230, Chile; karla.inostroza@ufrontera.cl (K.I.); nestor.sepulveda@ufrontera.cl (N.S.); 3 Instituto de Producción Animal, Facultad de Ciencias Agrarias y Alimentarias, Universidad Austral de Chile, P.O. Box 567, Valdivia 5110556, Chile; silvana.bravo@uach.cl; 4 Laboratorio de Fisiología y Biología Molecular Vegetal, Facultad de Cs. Agronómicas y Forestales, Center of Plant, Soil Interaction and Natural Resources Biotechnology, Scientific and Technological Bioresource Nucleus, Universidad de La Frontera, Casilla 54D, 1145 Temuco 4811230, Chile

**Keywords:** *Hymenophyllum plicatum*, *Hymenophyllum caudiculatum*, desiccation tolerant plant, filmy fern, lipid content, fatty acid profile, membrane integrity, very long chain polyunsaturated omega-3 fatty acids

## Abstract

Lipids are fundamental components of cell membranes and play a significant role in their integrity and fluidity. Alteration in lipid composition of membranes has been reported to be a major response to abiotic environmental stresses. This work was focused on the characterization of frond lipid composition and membrane integrity during a desiccation–rehydration cycle of two filmy fern species with contrasting desiccation tolerance: *Hymenophyllum caudiculatum* (less tolerant) and *Hymenophyllum plicatum* (more tolerant). The relative water content decreased without differences between species when both filmy ferns were subjected to desiccation. However, *H. plicatum* reached a higher relative water content than *H. caudiculatum* after rehydration. Fatty acids profiles showed the presence of a very long chain polyunsaturated fatty acid during the desiccation–rehydration cycle, with eicosatrienoic acid being the most abundant. Additionally, propidium iodide permeation staining and confocal microscopy demonstrated that, following the desiccation–rehydration cycle, *H. plicatum* exhibited a greater membrane integrity than *H. caudiculatum*. The lack of some very long chain fatty acids such as C22:1n9 and C24:1n9 in this species contrasting with *H. plicatum* may be associated with its lower membrane stability during the desiccation–rehydration cycle. This report provides the first insight into the fatty acid composition and dynamics of the membrane integrity of filmy ferns during a desiccation–rehydration cycle. This could potentially play a role in determining the different levels of desiccation tolerance and microhabitat preferences exhibited by Hymenophyllaceae species.

## 1. Introduction

Desiccation is severe water loss, when cellular water content becomes restricted to limits where even molecular hydration shells can be disturbed [1]. Therefore, desiccation represents severe stress for living organisms, causing numerous injuries at the cellular and molecular levels, such as biological membranes [2]. Water is essential to maintain the stability and integrity of cellular membranes. Understanding how some species preserve membrane functionality in tissues and cells under desiccation events is of remarkable importance [3].

Lipids play an important role in the maintenance of the structure and function of cells [4]. Fatty acids (FAs) in the lipid bilayers of cell membranes are responsible for much of its stability [5]. During water stress, a typical response is a decrease in membrane lipid content [3,6], and this behavior has been correlated with the inhibition of lipid biosynthesis [7,8]. 

Most plant tissues are vulnerable to dehydration. Under this situation, vegetative tissues are damaged and will die once the water content falls below critical values [9]. Desiccation tolerant organisms can equilibrate their water content with the air at low relative humidity (≤50%), reducing their metabolism and then, when relative humidity increases, they are able to reestablish normal metabolic activity [10]. Hymenophyllaceae, also known as the filmy fern family, comprise more than 600 species that are abundant in humid tropical and temperate forests [11,12]. Members of this family have been recognized as desiccation-tolerant plants [13,14]. Taxonomic, ecological and biochemical studies have been conducted on this group of plants [12,13,14,15,16,17], but lipid characterization is a topic that is still not addressed. However, studies carried out on other desiccation-tolerant species have reported changes and/or the presence of particular fatty acids when plants are exposed to desiccation–rehydration cycles. Decreases in lipid content has been reported in *Craterostigma plantagineum* [18], *Ramonda serbica* [19] and *Sporobolus stapfianus* [20], along with increases in the unsaturation levels of *R. serbica* [19] or the detection of large quantities of unsaturated linolenic acid in *Syntrichia ruralis* [21]. A recent study of *Xerophyta humilis* has reported the presence of very long chain fatty acids from 20 to 32 carbons [22]. 

Previous reports [12,16] have related the vertical distribution pattern exhibited by filmy ferns in a temperate rainforest in southern Chile with interspecific differences in desiccation tolerance. We hypothesized that such interspecific differences are associated with different levels of cell membrane stability among Hymenophyllaceae after a desiccation–rehydration cycle, which is related to the presence of unsaturated lipids with special features in those species exhibiting a greater desiccation tolerance. In order to test this hypothesis, the aim of this work was to characterize the lipid composition and membrane stability levels of two filmy fern species with contrasting desiccation tolerance and microhabitat preferences: *Hymenophyllum caudiculatum* Mart., less desiccation tolerant and restricted to shady or moist microsites in the lower forest strata; and *Hymenophyllum plicatum* Kaulf., more desiccation tolerant and inhabiting from the lowest to the upper canopy on exposed microsites in forests [12,16].

## 2. Results

### 2.1. Water and Lipid Content

Fronds of *H. caudiculatum* and *H. plicatum* remained at 85% and 88% of RWC respectively under well-watered conditions in the nursery. RWC of both species declined significantly, reaching 13% for *H. caudiculatum* and 14% for *H. plicatum* after irrigation was stopped. Finally, when irrigation was restored, both species increased their RWC, but they responded differentially; *H. plicatum* reached 75 % RWC recovering to a higher extent than *H. caudiculatum*, which only reached a 56% RWC upon rehydration (Table 1).

Lipid content in *H. plicatum* did not change significantly at all evaluated steps during the desiccation–rehydration cycle, showing a mean value of 28 mg g^−1^ dry weight. The lipid content of *H. caudiculatum* decreased during the desiccation–rehydration cycle. In the hydrated state, fronds had about 44% more lipids than in the rehydrated stage (Table 1).

### 2.2. Fatty Acids Fractions (SFA, MUFA, PUFA)

**Figure 1:**
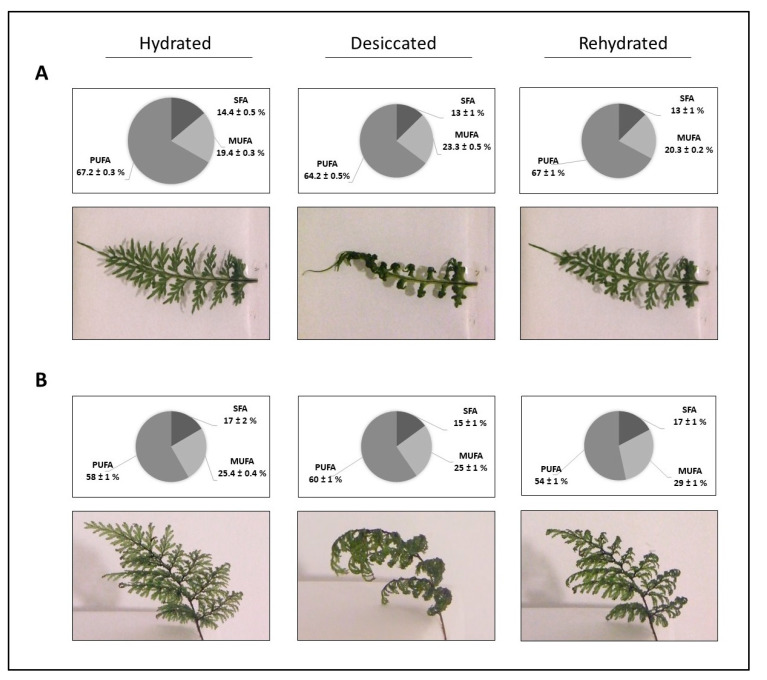
Fatty acid composition (%) and representative images of hydrated, desiccated and rehydrated fronds of two contrasting desiccation-tolerant filmy ferns subjected to a desiccation–rehydration cycle. (**A**) *Hymenophyllum caudiculatum*, less desiccation tolerant, and (**B**) *Hymenophyllum plicatum*, more desiccation tolerant. The corresponding values represent the mean of two biological replicates (± SE). Fatty acids nomenclature: Polyunsaturated (PUFA), monounsaturated (MUFA) and saturated (SFA).

FA compositions of *H. caudiculatum* and *H. plicatum* had similar distribution patterns at all frond water status. Among the FAs identified, polyunsaturated fatty acid (PUFA) was the most abundant type, followed by monounsaturated fatty acid (MUFA) and saturated fatty acid types (SFA) consecutively. In *H. caudiculatum*, SFA content was stable during the entire desiccation–rehydration cycle. Meanwhile, MUFA content was higher at the desiccated state than hydrated and rehydrated states in opposition to PUFA content that was lower at the desiccated state than hydrated and rehydrated. In *H. plicatum*, SFA contents did not show variations during the desiccation–rehydration cycle. However, MUFA content was higher at the re-hydrated state. Meanwhile the PUFA content, after its increase from the hydrated to the desiccated state, decreased again at the re-hydrated state (Figure 1). The content of “not identified” FAME was always a minor proportion, less than 8% in both species and at all frond water statuses.

Interspecific differences between FA composition were observed during the desiccation–rehydration cycle. *Hymenophyllum caudiculatum* showed a lower content of MUFA and SFA, but a higher content of PUFA than *H. plicatum* in all stages of the desiccation–rehydration cycle. Average contents (showed as percentage) of different FAs types in *H. caudiculatum* well-watered fronds were PUFA (67.2 ± 0.3%), MUFA (19.4 ± 0.3%) and SFA (14.4 ± 0.5%). Meanwhile, for *H. plicatum*, the FA contents under the same frond water status were PUFA (58 ± 1%), MUFA (25.4 ± 0.4%) and SFA (17 ± 2%) (Figure 1).

### 2.3. Fatty Acids Profile

The standard mixtures used in gas-chromatography allowed us to identify a diversity of 19 FAs in *H. plicatum* (Supplementary Figure S1) and 15 FAs in *H. caudiculatum* (Supplementary Figure S2). *H. caudiculatum* lacks C14:0, C18:2n6c, C22:6n3 and C24:1n9 FAs. Five major FAs were detected: cis-11,14,17-eicosatrienoic acid C20:3n3, 6,9,12-octadecatrienoic C18:3n6, cis-10-pentadecenoic acid C15:1, cis-9-octadecenoic C18:1n9c and octadecanoic acid C18:0 in all analyzed samples (Table 2). Interestingly, FA profiles fluctuated during the desiccation–rehydration cycle and these fluctuations were different for the two species. Despite such variations, a polyunsaturated very long chain fatty acid (VLCFA), cis-11,14,17-eicosatrienoic acid (C20:3n3), was the most abundant FA in both filmy ferns (Table 2). In detail, various changes were observed during the desiccation–rehydration cycle. Particularly, in *H. caudiculatum*, only four FAs corresponding to the 27% of all FAs identified at the hydrated state, showed significant variations when exposed to the desiccation–rehydration state (Table 2). These changes were detected also in major FA components; C15:1 (cis-10-pentadecanoic acid) and C18:3n6 (6,9,12-octadecatrienoic) considered between of the most abundant FA. In 6,9,12-octadecatrienoic (C18:3n6), the initial content decreased after desiccation and it was maintained low after rehydration. Octanoic acid (C8:0) exhibited an increase after desiccation and this higher content was maintained after rehydration. Cis-10-pentadecanoic acid (C15:1) on the other hand, after the desiccation step, exhibited a decrease in content at the rehydrated state to values similar to the initial content (well-hydrated fronds). Other FA contents increased significantly during desiccation and then decreased but remain slightly above initial levels such as cis-10-heptadecenoic acid (C17:1). When the corresponding contents were compared with the initial values, this FA was about 155% higher at the desiccated state and 17% higher at the rehydrated state (Table 2).

Fifteen fatty acids exhibited variations during the desiccation–rehydration cycle in *H. plicatum* (including four of the five most abundant FAs) which corresponded to 79% of the total fatty acids identified at the hydrated state (Table 2). Differences among the frond water status were found in cis-10-pentadecanoic acid (C15:1), cis-10-heptadecenoic acid (C17:1) and cis-4,7,10,13,16,19-docosahexaenoic (C22:6n3) specifically, these acids showed decreased contents at desiccated and rehydrated states. Cis-13-docosenoic acid (C22:1n9) and tetracosanoic acid (C24:0), appeared in desiccation state and was maintained during rehydration. Butanoic acid (C4:0), cis-9-octadecenoic (C18:1n9c) and 6,9,12-octadecatrienoic (C18:3n6) contents were not affected by desiccation, however their contents decreased upon rehydration. Cis-11,14,17-eicosatrienoic (C20:3n3) levels increased during the desiccated state, but in rehydrated state decreased back to a similar value as those observed in the hydrated state. Cis-8,11,14-eicosatrienoic (C20:3n6) decreased slightly during desiccation and at the rehydration state decreased drastically. Undecanoic acid (C11:0), tetradecanoic acid (C14:0) and hexadecanoic acid (C16:0) disappear at the desiccated state and then in the rehydrated state appeared with a slightly decrease. Hexanoic acid (C6:0) exhibited decreased contents after desiccation, which then recovered after rehydration. Cis-15-tetracosenoic acid (C24:1n9) appeared at the desiccated state and decreased their content during rehydration. The remaining FAs did not show significant differences between the hydration stages evaluated.

### 2.4. Membrane Integrity

**Figure 2:**
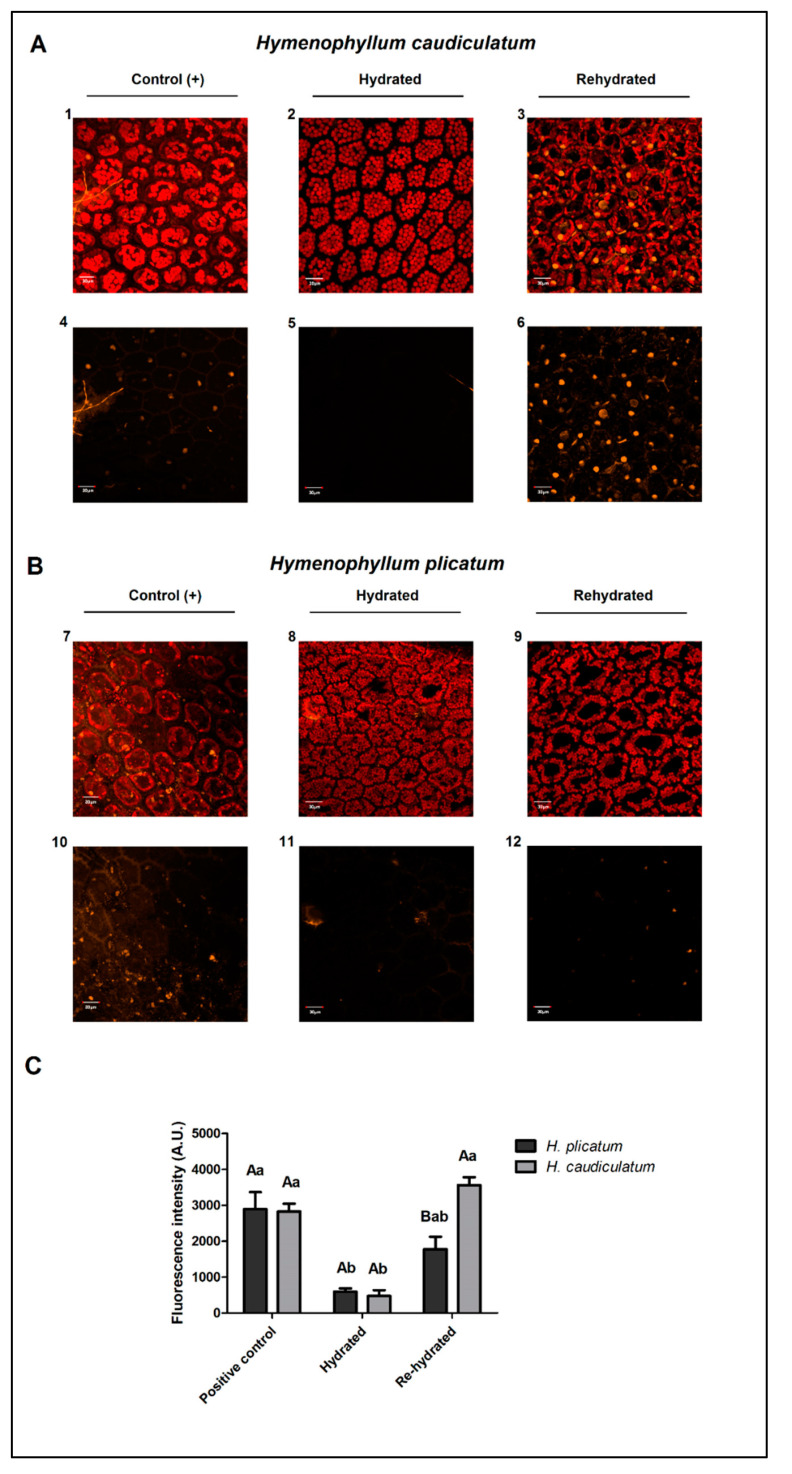
Changes in membrane integrity during a desiccation–rehydration cycle in two filmy fern species with contrasting desiccation tolerance: (**A**) *Hymenophyllum caudiculatum*, less desiccation tolerant, (**B**) *Hymenophyllum plicatum*, more desiccation tolerant, (**C**) Fluorescence intensity in arbitrary units for propidium iodide channel. Hydrated and rehydrated fronds of both species were stained with propidium iodide for 30 min and visualized by confocal laser scanning microscopy. Bars represent the mean ± SE of 5 areas of interest within each representative image of propidium iodide fluorescence. Lowercase letters indicate intraspecific differences among the three stages of water status, while uppercase letters indicate interspecific differences at each water status (Holm-Sidak method, *p* ≤ 0.01). Panels **A2**,**A3** and panels **B8**,**B9** are maximal projection of optical z-stacks of merged signal from propidium iodide fluorescence (orange) and internal control of chlorophyll fluorescence (red). Panels **A5**,**A6** and **B11**,**B12**, are maximal projections of optical z-stacks of propidium iodide fluorescence (orange). Panels **A1**–**A4** and **B7**–**B10** are from the positive stained control of permeabilized cells (cellulase enzyme plus PI in DMSO). Scale bars: 30 µm.

The integrity of the cell membrane was visualized by staining tissue with propidium iodide (PI), a fluorescent nucleic acid dye that enters only damaged cell membranes, producing an orange fluorescence [23]. At the full hydration condition, fronds of both species showed low fluorescence signals and low staining of the nucleus, indicating no damaged membranes in fully hydrated fronds (Figure 2, panels A, 2 and 5 and panels B, 8 and 11). However, at the rehydrated state, only the fronds of *H. caudiculatum* exhibited a high number of cells, with an increased orange fluorescence signal, particularly staining coming from the nucleus (Figure 2, panels A3 and panels A6), indicating that PI was able to enter the cell through damaged membranes caused by the desiccation–rehydration cycle.

## 3. Discussion

Water loss in filmy ferns (poikilohydric) is highly dependent on environmental conditions [24]. Leaf or frond RWC is an indicator of the internal water status, which is widely used for the evaluation of leaf hydration state or internal water deficit level during dehydration [25]. Our results indicate that the desiccation level reached by *H. caudiculatum* and *H. plicatum* fronds under the experimental conditions (nursery garden and laboratory), were similar. However, the rehydration step showed a higher water content recovery in fronds of *H. plicatum*. This result could be associated with greater desiccation tolerance considering that recovery of water content has been positively related with a reestablishment of metabolic activity such as respiration [26]. Previous work done with these same species described a higher desiccation tolerance in *H. plicatum* than *H. caudiculatum* [12], which is consistent with the present results. Similar results have been reported in *Bryum argenteum*, a desiccation tolerant moss, were almost a complete recovery of RWC was recorded after 10 min of rehydration [27]. Other research evaluating the response of *Selaginella lepidophylla*, a desiccation-tolerant moss and *Seleginella moellendorffii*, a desiccation susceptible specie, displayed a differential response to rehydration while *S. lepidophylla* recovered their RWC after 24 h of rehydration; *S. moellendorffi* cannot fully recover from desiccation [28].

A lipid remodeling is a common plant response to water deficit and to environmental stresses in general [29]. A reduction of total lipids has been observed in response to abiotic stress. Such behavior was only observed during the desiccation–rehydration cycle of *H. caudiculatum* (Table 1), the most vulnerable of these desiccation-tolerant filmy fern species. A decline in lipid content in both detached and non-detached leaves of *Sporobolus stapfianus*, another desiccation-tolerant plant, has been reported during desiccation. The successful recovery was associated with protective and/or repair mechanisms activated during the desiccation and rehydration processes in attached leaves [20]. The desiccation process in the present study was also done with attached leaves. Hence, decreased lipid contents observed in *H. caudiculatum* could be associated with the disability of this fern to fully recover its original frond hydration stage upon rehydration due to the reduction of the cell membrane integrity. This was corroborated by observations of a higher extent of staining of the cell nucleus by PI in rehydrated fronds of *H. caudiculatum* observed under confocal microscopy. This result suggests a higher extent of membrane damage by the desiccation–rehydration cycle in *H. caudiculatum* than in *H. plicatum*, which in contrast, exhibited negligible PI staining upon rehydration (Figure 2).

Plants are distinct from many eukaryotes organisms in their ability to produce a diversity of FA structures (>450) [30] and a little group of five FAs (C16:0, C18:0, C18:1, C18:2 and C18:3) occurs widely in membrane lipids denominated by “common fatty acids” [31]; however, “unusual fatty acids” include those that contain fewer than 16 or more than 18 carbons and these are often major components of seed oils plants [30]. The fatty acid biosynthesis has two major pathways in plants, the eukaryotic and prokaryotic in which fatty acids are synthesized in the plastid or endoplasmic reticulum, respectively [32]. VLCFAs biosynthesis is catalyzed by an alternating sequence of fatty acid desaturation and elongation of primary biosynthesis that requires three primary enzyme activities: Δ6-desaturase, Δ6-elongase and Δ5-desaturase present in the endoplasmic reticulum [33]. VLCFAs are incorporated into four major lipid pools (triacylglycerols, waxes, phospholipids, complex sphingolipids) [34]. Vegetative fronds (leaves), tissue fatty acids from filmy fern, were analyzed in this study. These filmy ferns lack cuticles and epicuticular waxes, so these VLCFA could only be part of triacylglycerols phospholipids and/or complex sphingolipids.

No prior reports were identified concerning the abundance of the unsaturated FA cis-11,14,17 eicosatrienoic (20:3n3) in the filmy fern’s family. This FA has been reported earlier in marine species, such as *Sirodotia* sp. [35] or *Eucheuma cottonii* [36], both species belonging to rhodophyta phylum. However, others VLCFA (C20:2n6, C20:3n6, C20:4n6, C20:4n3 and C20:5n3) have also been established in ferns [37] or in the moss *Physcomitrella patens* [38]. An interesting report done in *Xerophyta humilis* informed the presence of VLCFA and related them with growing environmental conditions (high evaporative demand). The authors proposed a possible role of VLCFA in the regulation of water loss [22]. Another probable role of VLCFAs could be the minimization of mechanical stress produced by the desiccation process, and how they may promote the curvature of lipid bilayers because of large hydrophobic tails [39,40]. Interestingly, *H. plicatum* does not show the highest content in VLCFAs but exhibits a diversity of other minor VLCFAs, 8,11,14-eicosatrienoic acid (C20:3n6) and 4,7,10,13,16,19-docosahexaenoic acid (C22:6n3) in a higher content than *H. caudiculatum* and others only detected in *H. plicatum* 13-docosenoic acid (C22:1n9) and 15-tetracosenoic acid (C24:1n9). This diversity of VLCFAs observed in *H. plicatum* could be related to its best performance of membrane integrity observed upon desiccation rehydration of this filmy fern species. However, more direct proof needs to be established.

Concerning FA profiles during the desiccation–rehydration cycle, alterations on the unsaturation levels are traditionally related to changes in the membrane bilayer thickness and fluidity so a decrease in fatty acid unsaturation results in a decrease in membrane fluidity [41,42]. SFAs fractions did not show significant changes both in *H. caudiculatum* and *H. plicatum*. Nevertheless, PUFA fraction showed a slight increase from 58% to 60% during desiccation in *H. plicatum*, the most desiccation tolerant species analyzed, while a small decrease in PUFA fraction was observed (from 67% to 64%) in *H. caudiculatum*. However, the PUFA fraction was proportionally higher in *H. caudiculatum*, therefore, the evidence does not sustain a relationship between unsaturation of FA, membrane fluidity and stability during desiccation–rehydration in filmy ferns. In other resurrection plants, a dissimilar response has been observed in fatty acid profile during the desiccation–rehydration cycle; while in *Ramonda serbica* an increase in C18:1 was distinguished, a slight decline in C18:3 was registered and a significant decline in C18:2 was observed [43]. In *Paraisometrum mileense*, an increase in the level of unsaturation in all glycerolipid species was reported [44]. Therefore, there is still some controversy regarding what the real role of lipids is, and whether their unsaturation degree affects membrane stability in desiccation tolerance.

Higher levels of saturated FAs (C16:0 and C18:0) may increase the rigidity of cell membranes because of the linearity of FA arrangements in the membrane phospholipid phase [45]. Fronds of *H. caudiculatum* exhibited lower amounts of C16:0 and C18:0 FAs than *H. plicatum*, besides showing a tendency to decrease during the desiccation–rehydration cycle. In *Syntricia ruralis* and *Selaginella lepidophylla*, other desiccation tolerant plants, it has been reported that during desiccation cell membranes did not undergo alterations in the saturation/unsaturation levels [46]. Nonetheless, for desiccation-tolerant plants, the cell membrane stability is essential for the successful tolerance of a desiccation process. Our results from these filmy fern species support the idea that membrane stability during desiccation–rehydration does not rely merely on membrane fluidity due to the unsaturation of fatty acids. It may rely on the presence of particular minor VLCFAs such as 8,11,14-eicosatrienoic acid (C20:3n6) and 4,7,10,13,16,19-docosahexaenoic acid (C22:6n3), which exhibited higher contents in *H. plicatum* than *H. caudiculatum* and others only detected in *H. plicatum* such as 13-docosenoic acid (C22:1n9) and 15-tetracosenoic acid (C24:1n9) fatty acids. Another alternative explanation besides this lipid profile, is the interaction of membranes with other biochemical membrane stabilizers such as the amphipathic LEA proteins or sugar-alcohols, such as those observed in *Xerophyta schlechteri* [47] or *Pitcairnia burchellii* [48]. It is likely that a more complex hypothesis should be invoked to explain our observations regarding differences in membrane stability observed between these two filmy ferns species (Figure 2) contrasting in their desiccation tolerance.

## 4. Materials and Methods

### 4.1. Plant Material and Maintenance Conditions

Plant material was collected from a temperate rainforest in Southern Chile (Katalapi Park, Pto Montt, 41°31′ S, 72°45′ W). The samples consisted of the living individuals of two filmy ferns species with contrasting desiccation tolerance and microhabitat preferences: *Hymenophyllum caudiculatum* Mart. var. *productum* (K. Presl) C. Chr., less desiccation tolerant and restricted to shady or moist microsites in the lower forest strata; and *Hymenophyllum plicatum* Kaulf., more desiccation tolerant inhabiting the lower to the upper canopy and/or exposed microsites in the forest [12,16]. Both epiphytes species were obtained up to 1.5 m above the lower forest strata with its natural substrate consisting of small pieces of bark and/or host-tree brunches, each of these pieces was considered as a biological replicate. After their collection, samples were carefully transferred to the Laboratory of Plant Physiology and Molecular Biology nursery garden located at Universidad de La Frontera (Temuco, Chile), where they were maintained under growing conditions that mimicked their natural environment. Specifically, filmy ferns with their natural substrate were placed in an artificial wall manufactured with an Acma stainless steel mesh covered with three layers of burlap to keep a moist surface upon watering. Light intensity (25–30 µmol photons m^−2^ s^−1^) and water conditions (95% RH) were respectively provided by a black mesh and an automatic irrigation system composed of a fog type irrigation micro-sprinklers, which irrigated the ferns for 2 min every 2 h.

### 4.2. Treatments and Sample Collection 

Collected samples were acclimated for 4 weeks before the beginning of dehydration experiments. Three frond water status points were defined for evaluation, based on fronds relative to water content (hydrated, desiccated, and rehydrated). Hydrated fronds were collected immediately after the first step of watering in the early morning, and then the irrigation system was stopped and after 10 days of watering restriction, the desiccated fronds were collected. Later on, plants were rehydrated by 3 steps (2 min each) of mist irrigation with an interval of 30 min. Fronds coming from three different plant individuals were randomly collected from each treatment (hydrated, desiccated and rehydrated) and pooled into three samples (about 1500 mg each) which were quickly immersed in liquid nitrogen and stored at −80 °C in a freezer until analyses. Relative water content (RWC) was calculated previously for immersion in liquid nitrogen in sub-samples of each collected treatment as previously described [12], using three biological replicates for each treatment and filmy fern species under study.

### 4.3. Leaf Lipid Extraction

Frozen frond samples stored at −80 °C were lyophilized to remove all residual water which may have interfered in the lipid extraction. Lipids contained in each sample were extracted through a miniaturized Bligh-Dyer method [49]. Lyophilized fronds (100 mg per treatment) were extracted in a two-step protocol; first using a monophasic ternary system of methanol:chloroform:phosphate buffer pH 7.4 (2.5:5:2 *v*/*v*/*v*) and then converted to a biphasic state by dilution with additional chloroform and phosphate buffer (1:1 *v*/*v*). To purify the lipid extract and to remove the interfering pigments (e.g., chlorophyll), samples were loaded into an activated charcoal column. The mass of solvent-free lipids was gravimetrically determined by triplicate. For fatty acid analysis, a new extraction of two pooled samples (each containing fronds from 3 different individual plants) was done for each treatment, following the same steps described above. The cleaned extract was exposed under a nitrogen stream to evaporate and remove the organic solvents used in the extraction procedure.

### 4.4. Fatty Acid Analysis

Cleaned lipid extracts were stored overnight at −20 °C in the dark and were subsequently used for methyl ester preparation. For the formation of fatty acid methyl esters (FAMEs), 800 µL of n-hexane and 1.3 mL of 2N methanolic KOH (Merck, Germany) were added to each sample and then shaken magnetically for 30 min. The upper phase containing FAMEs was placed in a centrifuge tube and 0.5 g of Na_2_SO_4_ (Merck, Germany) was added. After centrifugation at 2000× *g* for 5 min at room temperature, the supernatant was recovered in vials for chromatography. The FAMEs obtained were analyzed by gas chromatography in a Clarus 500 chromatograph (Perkin Elmer, USA) equipped with a flame ionization detector (FID), autosampler, split injection and a fused silica capillary column SP^TM^ 2380 (60 m × 0.25 mm × 0.2 μm film thickness) (Supelco, Bellefonte, PA, USA). Later, 1 µL of FAMEs extract was injected under increasing chromatograph temperature conditions. The initial oven temperature was 150 °C (held for 1 min). This was subsequently increased to 168 °C at a rate of 1 °C min^−1^ (held for 11 min), and to 230 °C at 6 °C min^−1^ (held for 8 min). The temperature of the injector and FID were 250 °C and nitrogen was used as the carrier gas at a flow rate of 1 mL min^−1^. Individual FAMEs were identified by comparing them to a standard mixture of 37 components FAME Mix C4-C24 (Supelco, USA). The relative mass of each FAME was calculated using the response factor mass/peak area for each FAME standard following the recommendations of ISO 15304:2002 [50].

### 4.5. Membrane Integrity Evaluation

To assess the membrane integrity during a desiccation–rehydration cycle, hydrated and rehydrated fronds were observed under a confocal laser scanning microscope (CLSM) Olympus Fluoview 1000 (Olympus, Japan). The experimental procedure consisted in sampling hydrated fronds normally irrigated at the nursery garden, which were separated into two groups: one group was immediately stained for membranes visualization at the hydrated state and the other group was desiccated for 24 h under controlled laboratory conditions (22 ± 5 °C, 75 ± 5 HR and darkness), and then rehydrated for 24 h for membrane visualization at the rehydrated state. Samples of both treatments were stained using propidium iodide (PI) dissolved in phosphate saline buffer at 100 mM final concentration. Frond incubation was done during 30 min at room temperature in the dark. In parallel, another group of fronds was treated with a cellulase enzyme using PI dissolved in DMSO also at 100 mM final concentration as the positive control for the experimental staining procedure. The fluorescence intensity of PI was registered to a wavelength excitation/emission 546 nm/590 nm and chlorophyll autofluorescence was used as an internal control (wavelength excitation/emission 633 nm/650–750 nm). The fluorescent intensity was calculated by the software FV10-ASW 2.0 and Z-stack images were acquired and rendered using the F10-ASW 2.0 Fluoview software.

### 4.6. Statistical Analysis 

Data of lipid, water contents (Table 1) and fluorescence intensity (Figure 2C) were analyzed by two-way ANOVA, using the statistical program SigmaStat 3.5 (Systat Software Inc., Chicago, IL, USA). Normality of the data was confirmed before the ANOVA test. Additionally, a post-hoc Holm–Sidak method analysis and mean comparisons were done for those variables with an ANOVA *p* value less than 0.01 of significance. Fatty acid contents (Table 2, Figure 1) are exhibited using descriptive statistics.

## 5. Conclusions

The present study, which is the first of its type in two filmy ferns with contrasting desiccation tolerance, provides a content of lipid and FA profiles along with the variations during a desiccation–rehydration cycle.

Both species present a very long chain polyunsaturated cis-11,14,17-eicosatrienoic acid (C20:3n3) at a very high proportion, around 30% of total fatty acid. Significant differences were found in lipid content and RWC. However, the saturation/unsaturation proportion of FAs in *H. caudiculatum* versus *H. plicatum* does not show great differences. Interestingly, some differences in fatty acid profiles was found between these species, such as the presence of 13-docosenoic acid (C22:1n9) and 15-tetracosenoic acid (C24:1n9) which were only detected in the most desiccation-tolerant filmy fern species. Some of these differences could be associated with the contrasting desiccation tolerance previously reported for these filmy fern species. However, deeper physiological evaluation is needed to understand how much of the variation of these parameters could determine different levels of desiccation tolerance.

## Figures and Tables

**Table 1:** Changes in relative water content (RWC) and lipid content during the three stages of a desiccation–rehydration cycle (hydrated, desiccated, rehydrated) in two filmy ferns species with contrasting desiccation tolerance: *Hymenophyllum caudiculatum*, less desiccation tolerant and *Hymenophyllum plicatum*, more desiccation tolerant.

	*H. caudiculatum*			*H. plicatum*		
	Hydrated	Desiccated	Rehydrated	Hydrated	Desiccated	Rehydrated
**RWC (%)**	85 ± 1 ^Aa^	13 ± 1 ^Ac^	56 ± 5 ^Bb^	88 ± 1 ^Aa^	14 ± 2 ^Ac^	75 ± 2 ^Ab^
**Lipid Content (mg g^−1^ Dry Weight)**	36 ± 2 ^Aa^	29 ± 2 ^Aab^	20 ± 3 ^Ab^	29 ± 2 ^Aa^	30 ± 4 ^Aa^	26 ± 1 ^Aa^

Values represent the mean of three biological replicates (± SE). Lowercase letters indicate intraspecific differences among the three stages of water status; uppercase letters indicate interspecific differences at each water status (Holm-Sidak method, *p* ≤ 0.01).

**Table 2:** Mean mass fraction (µg g^−1^ Dry Weight) of individual fatty acid changes in the hydrated, desiccated and rehydrated fronds of two contrasting desiccation-tolerant filmy ferns subjected to a desiccation–rehydration cycle. *Hymenophyllum caudiculatum*, less desiccation tolerant, and *Hymenophyllum plicatum*, more desiccation tolerant.

Fatty Acid	*H. caudiculatum*			*H. plicatum*		
	Hydrated	Desiccated	Rehydrated	Hydrated	Desiccated	Rehydrated
**C4:0**	25 ± 3	18 ± 2	14 ± 2	16 ± 3	19 ± 2	6 ± 1
**C6:0**	8 ± 1	9 ± 1	7 ± 1	7 ± 1	2.4 ± 0.2	4.6 ± 0.4
**C8:0**	2.7 ± 0.2	4.6 ± 0.1	4.1 ± 0.1	3.3 ± 0.5	2.3 ± 0.2	2.6 ± 0.2
**C11:0**	5.8 ± 0.5	5.5 ± 0.4	3.3 ± 0.4	3.3 ± 0.2	n.d.	2.5 ± 0.2
**C14:0**	n.d.	n.d.	n.d.	4 ± 2	n.d.	2.0 ± 0.2
**C15:1**	64 ± 2	72 ± 3	55 ± 8	77 ± 6	38 ± 1	44 ± 2
**C16:0**	3 ± 1	3 ± 1	1.4 ± 0.4	7± 3	n.d.	5 ± 1
**C17:1**	9.4 ± 0.4	24 ± 1	11 ± 1	14 ± 1	8 ± 1	8.1 ± 0.5
**C18:0**	25 ± 3	20 ± 1	17 ± 2	42 ± 4	30 ± 2	33 ± 1
**C18:1n9c**	47 ± 1	48 ± 2	39 ± 7	57 ± 5	64 ± 1	45 ± 3
**C18:2n6c**	n.d.	n.d.	n.d.	12 ± 1	10 ± 1	12 ± 2
**C18:3n6**	171 ± 2	150 ± 4	128 ± 26	124 ± 15	100 ± 2	64 ± 6
**C21:0**	10 ± 1	13 ± 2	10 ± 2	13 ± 1	18 ± 3	9 ± 1
**C20:3n3**	218 ± 2	234 ± 3	201 ± 33	141 ± 11	174 ± 4	109 ± 6
**C20:3n6**	18.0 ± 0.3	16 ± 1	11± 2	27 ± 3	21 ± 1	11 ± 1
**C22:1n9**	n.d.	n.d.	n.d.	n.d.	13 ± 1	10 ± 1
**C22:6n3**	9 ± 2	11 ± 1	10 ± 2	41 ± 2	8 ± 1	5.7 ± 0.3
**C24:0**	5.6 ± 0.2	6.9 ± 0.1	6 ± 1	n.d.	2 ± 1	1.1 ± 0.1
**C24:1n9**	n.d.	n.d.	n.d.	n.d.	10 ± 7	4.0 ± 0.5

The values represent the arithmetic mean of two biological replicates (± SE). n.d., Not detectable.

## Supplementary Materials

The following are available online at https://www.mdpi.com/2223-7747/9/11/1431/s1, Figure S1: Representative chromatogram of *H. plicatum* during desiccation–rehydration cycle. Figure S2: Representative chromatogram of *H. caudiculatum* during desiccation–rehydration cycle.
