# Supplementary figures and images for: Exploratory Study of Fatty Acid Profile in Two Filmy Ferns with Contrasting Desiccation Tolerance Reveal the Production of Very Long Chain Polyunsaturated Omega-3 Fatty Acids
Source: Plants (Basel). 2020 Oct 24;9(11):1431. doi: 10.3390/plants9111431 (PMC7692210; doi:10.3390/plants9111431)

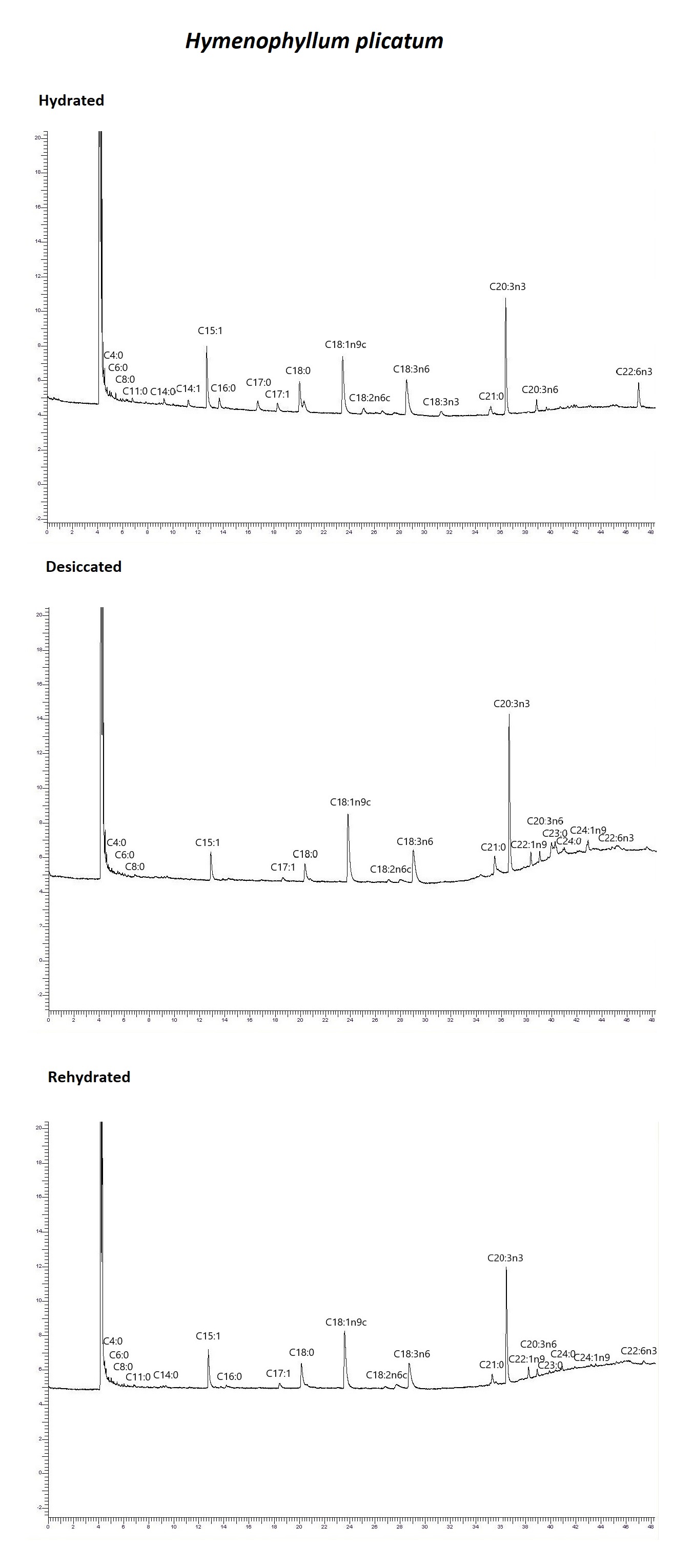

Supplement: Supplementary file 1 [file plants-09-01431-s001.zip › sup/S1 Plicatum.jpg]

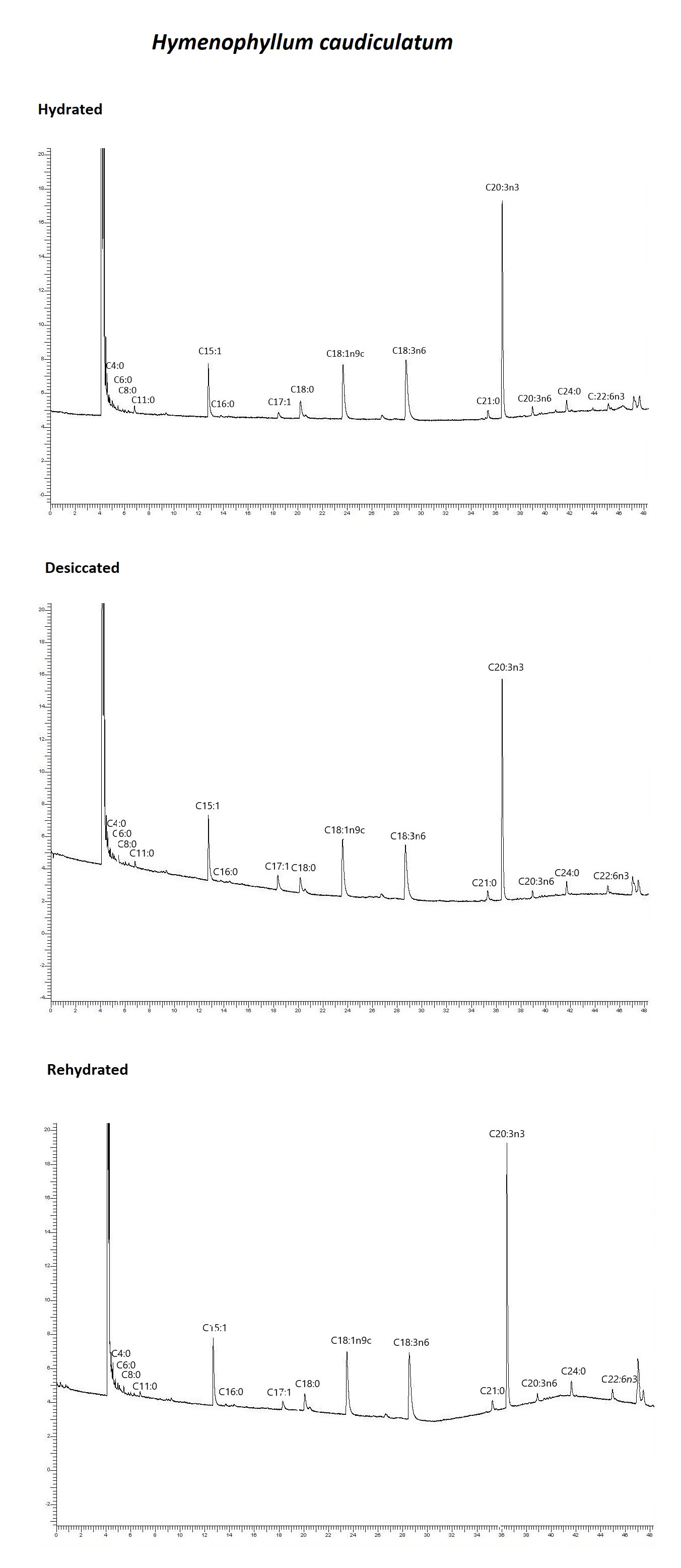

Supplement: Supplementary file 1 [file plants-09-01431-s001.zip › sup/S2 CAUDI.jpg]
